# Lower tumour burden and better overall survival in melanoma patients with regional lymph node metastases and negative preoperative ultrasound

**DOI:** 10.2478/v10019-011-0028-1

**Published:** 2011-09-22

**Authors:** Gasper Pilko, Janez Zgajnar, Maja Music, Marko Hocevar

**Affiliations:** 1 Institute of Oncology Ljubljana, Department of Surgical Oncology, Slovenia; 2 Institute of Oncology Ljubljana, Department of Radiology, Ljubljana, Slovenia

**Keywords:** melanoma, ultrasound, tumour burden, overall survival

## Abstract

**Background:**

The purpose of the study was to evaluate the ability of ultrasound (US) and fine needle aspiration biopsy (FNAB) in reducing the number of melanoma patients requiring a sentinel node biopsy (SNB); to compare the amount of metastatic disease in regional lymph nodes in SNB candidates with clinically uninvolved lymph nodes and of those with US uninvolved lymph nodes; and to compare the overall survival (OS) of both groups.

**Methods:**

Between 2000 and 2007, a SNB was successfully performed in 707 patients with melanoma. The preoperative US of the regional lymph node basins was performed in 405 SNB candidates. In 14 of these patients, the US-guided FNAB was positive and they proceeded directly to lymph node dissection. In 391 patients, the preoperative US was either negative (343 patients) or suspicious (48 patients) (US group). In the remaining 316 patients the preoperative US was not performed (non-US group).

**Results:**

The proportion of macrometastatic sentinel lymph nodes (SN), number of metastatic lymph nodes per patient and proportion of nonsentinel lymph node metastases were found to be lower in the US group compared to the non-US group. The smaller tumour burden of the US group was reflected in a significantly better OS of patients with SN metastases.

**Conclusions:**

The preoperative US of regional lymph nodes spares some patients with melanoma from undergoing a SNB. Patients with regional metastases and a negative preoperative US have a significantly lower tumour burden in comparison to those with clinically negative lymph nodes, which is also reflected in a better OS.

## Introduction

Lymph node status is the most important prognostic factor in patients with cutaneous melanoma.^1^,^2^ The presence of regional lymph node metastases reduces the 10-year survival rate by 20% to 50%, compared to patients with no lymph node metastases at the time of diagnosis.^2^ For this reason, an accurate knowledge of the nodal status at the time of primary melanoma diagnosis is critical, both to guide treatment and to provide patients with a reliable estimate of their prognosis.^3^,^4^

Over the past decade, the sentinel lymph node biopsy (SNB) has become the method of choice for the staging of regional lymph nodes in patients with melanoma and also other cancers.^5^–^7^ The procedure provides valuable prognostic information and facilitates early therapeutic lymphadenectomy in patients with clinically occult regional metastases, with only a slight increase in costs.^8^ However, on the other hand, the SNB procedure is time consuming, logistically demanding, and a second operation is required in cases where the SNB is positive.^9^,^10^ On the basis of these data, efforts were made to assess other less invasive techniques. Studies have demonstrated that a preoperative high resolution ultrasound (US) combined with a fine needle aspiration biopsy (FNAB) reliably detects lymph node metastases which are larger than 2–4 mm.^9^,^11^–^16^ Patients with a positive FNAB can proceed directly to lymph node dissection instead of undergoing an initial SNB. Therefore US, combined with FNAB may prevent unnecessary anaesthesia and surgical management as well as reduce costs. However, until now, the impact of the preoperative US examination of clinically negative regional lymph nodes on the amount of detected metastatic disease and, therefore, the prognosis has not yet been assessed. We can only assume that the tumour burden is significantly lower in patients with US negative lymph nodes.

The aims of this study were to evaluate the ability of a US and a US-guided FNAB to reduce the number of patients requiring a second surgical procedure and to compare the amount of metastatic disease in regional lymph nodes in SNB candidates with clinically uninvolved lymph nodes (non-US group) and of those with US uninvolved lymph nodes (US group). The overall survival (OS) of both groups of patients was also compared.

## Patients and methods

### Patients

Between 2000 and 2007, a SNB was successfully performed in 707 patients with cutaneous melanoma at the Institute of Oncology Ljubljana, Slovenia. All the patients had clinically negative lymph nodes and none of the patients included exhibited clinical evidence of systemic disease at the time of surgery. The preoperative work-up consisted of obtaining a thorough medical history, a clinical examination with an emphasis on the skin and regional lymph nodes, and a serum S-100 protein test. Additional imaging scans (CT, US or MR) were only taken when different clinical signs and/or symptoms were present.

The preoperative US of the regional lymph node basins was carried out in 405 SNB candidates. In 14 of these patients, the US-guided FNAB was positive and they proceeded directly to lymph node dissection. In 343 patients, the preoperative US of the regional lymph node basins was negative. In an additional 48 patients, the US of the regional lymph node basins was suspicious for lymph node metastases. In 24 of those patients, a US-guided FNAB was performed, but tested negative for malignancy. In the remaining 24 patients with a suspicious US, a US-guided FNAB was not performed due to technical difficulties (a small or inaccessible target). In the additional 316 patients who underwent SNB, a preoperative US of the regional lymph node basins was not performed.

The analysis therefore included 343 patients with a negative preoperative US, 48 patients with a suspicious preoperative US, and 316 patients with clinically uninvolved lymph nodes (non-US group).

The data on patients’ gender, age, tumour pathomorphological characteristics, locoregional control, disease free survival (DFS) and OS were collected.

All patients were routinely followed-up at the Institute’s outpatient department every 3–4 months during the first 2 years, every 6 months between the third and fifth years, and then annually thereafter. The follow-up consisted of obtaining a thorough medical history, a clinical examination with an emphasis on the skin and regional lymph nodes, and a serum S-100 protein test. Additional imaging scans (CT, US or MR) were only taken when different clinical signs and/or symptoms were present.

Recurrences were scored as local, regional, distant subcutaneous or visceral metastases.

The study was reviewed and approved by the Institutional Medical Ethics Committee.

### Preoperative US procedure

A preoperative US examination of regional lymph nodes was carried out, as described in detail elsewhere.^12^ The US was performed before lymphoscintigraphy and all possible nodal basins were examined according to Sappey’s lymphatic anatomy, (*e.g*. both axillas in trunk melanomas located medially more than 5 cm above the umbilicus). Examinations were carried out by an oncologically dedicated radiologist using a linear array transducer, a small parts probe of 12 and 15 MHz (Power Vision 8000, Toshiba Corporation, Ottawara, Japan). The US features considered as suspicious or malignant were a rounded appearance of the lymph node (changed from long to short diameter), the loss of the hilar echogenic reflex and a deformed radial nodal vascularity.^17^,^18^ In patients with US suspicious or malignant lymph nodes, a US-guided FNAB was performed where technically possible.

### Sentinel lymph node procedure

The triple technique was used for sentinel node (SN) identification, as already published.^19^ Excised SNs were bisected along the long axis, fixed in 10% formalin and embedded in paraffin. A pair of sections (sections 1 and 2) was made from each block; the first was stained with H&E and the second for S-100 protein by immunohistochemistry (IHC). If the initial review of these sections was negative, six additional consecutive sections were made (sections 3 to 8). Sections 3, 5 and 7 were stained with H&E, sections 4 and 8 for S-100 protein, and section 6 for HMB45. IHC stainings were performed using the avidin-biotin-peroxidase complex method with commercially obtained antibodies – S100 and HMB45 (Dako, Glostrup, Denmark).

In order to estimate the SN tumour burden, the maximum diameter of the largest lesion was used, according to Rotterdam criteria.^20^ We arbitrarily divided patients up into those with a positive SN on those with SN metastases with a diameter of 0.2 mm or less, those with a diameter of between 0.2 mm and 2.0 mm, and those with SN metastases with a diameter of more than 2.0 mm.

An SNB was indicated for patients with melanoma with a minimum Breslow thickness of 1.00 mm, or in the case of a Breslow thickness of less than 1 mm if the Clark level was IV/V, or if ulceration was present. There was no age restriction on performing a SNB.

### Statistical analysis

For univariate analysis, t-test, Mann-Whitney test and contingency tables were used. All factors that showed a statistically significant correlation on univariate analysis were included in multivariate analysis. For multivariate analysis, logistic regression was used. Survival curves were calculated by Kaplan-Meier’s method.

The differences were considered statistically significant if the p values were less than 0.05. Software package SPSS 15.0 for Windows was used.

## Results

### The ability of US and US-guided FNAB in reducing the number of patients requiring a SNB

In 14 SNB candidates, the US-guided FNAB was positive for metastases and they proceeded directly to lymph node dissection.

Of them, 7 SNs were located in the inguinal region, 6 in the axilla and one SN was located in the neck.

Median diameter of SN metastases identified with US-guided FNAB was 25.0 mm (range 4.5–50.0 mm).

Their 5-year OS was 41%.

### Patients’, tumour and lymph node characteristics and comparison of the tumour burden between the groups

SNB was successfully performed in 707 patients (324 men and 383 women). The median age of all the patients was 56 years (range 7–93 years). The median Breslow thickness of all the patients’ primary tumours was 2.1 mm (range 0.5–18.0 mm).

Altogether, 1456 SNs were removed (median 2/patient, range 1–10). The SNB was positive in 160 patients and completion lymph node dissection (CLND) was performed on all of them.

Of the patients with a positive SNB, 63 had SN metastases with a diameter of more than 2.0 mm, 78 had SN metastases with a diameter of between 0.2 mm and 2.0 mm, and 19 had SN metastases with a diameter of 0.2 mm or less. After CLND, 129 out of 160 patients had no additional metastases in nonsentinel lymph nodes (NSN), while 31 patients had one or more additional metastases in the CLND specimen.

Three groups of patients (non-US group, US negative and US suspicious) were well matched with no statistically significant difference in the patients’ age, tumour thickness according to Breslow, or the presence/absence of ulceration (Table 1). However, there were statistically important differences in the proportion of patients with SN metastases with a diameter of more than 2.0 mm and the total number of metastatic lymph nodes per patient (Table 1).

### Survival analysis

The median follow-up was 42 months (range 1–132 months). During this time, the disease recurred in 157 patients; in 43 of these patients, a local recurrence was diagnosed; regional lymph node metastases and distant metastases were diagnosed in 57 and 85 patients, respectively. The 5-year DFS for the whole group was 80%.

Of the patients included in the analysis, 106 died of melanoma and 18 died from other causes. The 5-year OS of all patients was 86.6%.

There was a significant difference in the 5-year OS between SN-positive and SN-negative patients – 65% compared to 92.6% (p<0.001).

**FIGURE 1:**
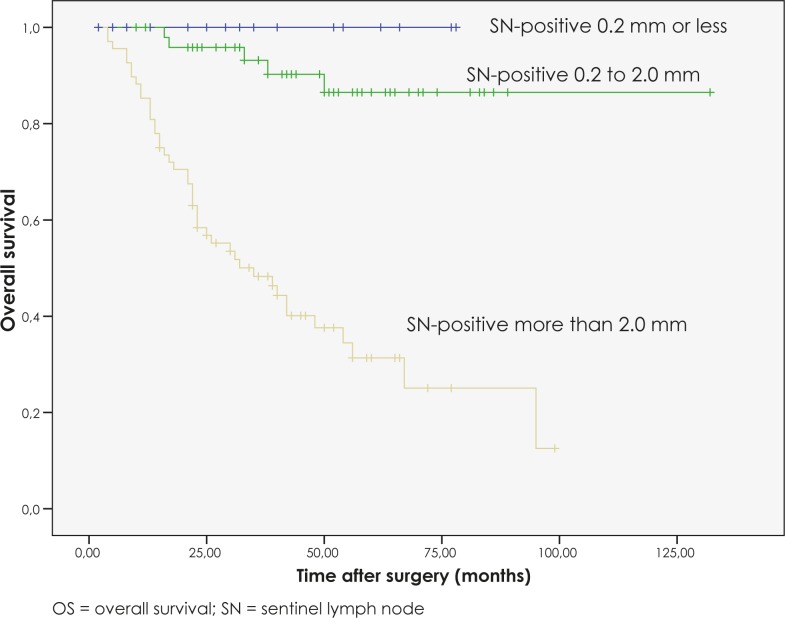
OS curves for SN-positive patients according to size of SN metastasis.

For those patients with a positive SNB, there was a significant difference in the 5-year OS between those patients with SN metastases with a diameter of 0.2 mm or less, patients with SN metastases with a diameter of between 0.2 mm and 2.0 mm, and those patients with SN metastases with a diameter of more than 2.0 mm (p<0.001). None of the patients with SN metastases with a diameter of 0.2 mm or less died from disease during the follow-up. Patients with SN metastases with a diameter of between 0.2 mm and 2.0 mm had a 5-year OS of 90%, whereas patients with SN metastases with a diameter of more than 2.0 mm had a 5-year OS of 39.7% (Figure 1).

In the whole group of the patients, there was a non-significant difference in OS between patients with a negative US, those with a suspicious US and the non-US group of patients (p=0.513), with a 5-year OS of 88%, 81% and 85%, respectively.

Among the patients with positive SNs, the patients from the non-US group had a significantly worse OS than those with negative and suspicious US, with a 5-year OS of 43%, 70% and 65%, respectively (p=0.013).

Since there was no significant difference in OS between those patients with a negative US and those with a suspicious US (p=0.837), we decided to merge both groups into one (US group) and compare its tumour burden to the non-US group of patients.

The proportion of patients with SN metastases with a diameter of more than 2.0 mm (31 out of 101 versus 32 out of 59, p=0.007), the total number of metastatic lymph nodes per patient (1.2 versus 1.7, p=0.008) and the proportion of NSN metastases (15 out of 101 versus 16 out of 59, p=0.047) were found to be lower in the US group compared to the non-US group (Table 2).

**FIGURE 2:**
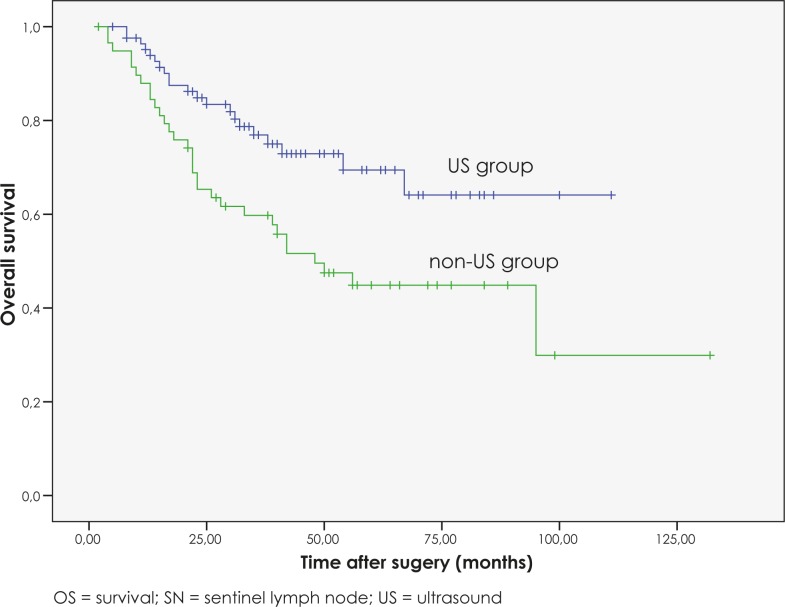
OS curves for SN-positive patients according to preformed preoperative US.

Among the patients with positive SNs, the patients in the US group had a significantly better OS than those in the non-US group, with a 25% difference in the 5-year OS (p=0.003) (Figure 2).

### Factors correlated with the presence of SN metastases with a diameter of more than 2.0 mm on univariate and multivariate analysis

The factors which correlated with the presence of SN metastases with a diameter of more than 2.0 mm on univariate and multivariate analysis were preoperative US not preformed (p=0.002), the presence of ulceration (p=0.025) and the number of positive SNs (p=0.017) (Table 3).

### Factors correlated with the presence of NSN metastases on univariate and multivariate analysis

The factors which correlated with the presence of NSN metastases on univariate and multivariate analysis were the Breslow thickness (p=0.004), the size of the SN metastasis (p=0.003) and the number of removed SNs (p=0.04) (Table 4).

## Discussion

In our study, 3.5% of the patients on whom a US was performed did not proceed to SNB because the US-guided FNAB proved lymph node metastases preoperatively. This is in line with several studies conducted which have demonstrated that a high-resolution US is a more sensitive and specific alternative to physical examination when it comes to the detection of lymph node metastases.^21^–^25^ The combination of a US and FNAB eliminated the need for a SNB in 2.3% to 16% of the patients in published studies.^9^,^11^–^15^,^26^,^27^ Such a wide range can be explained partly by the different proportion of patients with a small tumour burden in their lymph nodes that were included in each study. This relatively low number of patients spared from undergoing an SNB in our study could be explained by the high proportion of patients with SN metastases with a diameter of 2.0 mm or less. The smallest metastatic deposit detected by a US in our study was 4.5 mm. These results are in line with the results of Starritt and colleagues^27^ which concluded that a preoperative US can detect metastatic melanoma deposits as small as approximately 4.5 mm in diameter. Similar results were also demonstrated by Sibon and colleagues^15^ who failed to detect metastatic deposits of less than 5 mm in diameter. However, according to the results of Rossi^14^ and Kunte^9^, the threshold lies way below 5 mm or 4.5 mm, at approximately 2 mm.

We can assume that the preoperative US of the regional lymph node not only spares some patients from undergoing a SNB procedure, but because of its higher sensitivity in comparison to palpation, it should also reflect in the smaller amount of the tumour burden detected in regional lymph nodes and, as such, also in the patient’s survival. A previous study conducted by Zgajnar and colleagues from our institute revealed that patients with early breast cancer and US uninvolved axillary lymph nodes have a significantly lower tumour burden in the axillary lymph nodes compared to those with only clinically uninvolved lymph nodes.^28^ However, to the best of our knowledge, this is the first study investigating the impact of a preoperative US examination of regional lymph nodes on the amount of the detected tumour burden in regional lymph nodes in clinically node negative melanoma patients.

Indeed, the present study clearly demonstrates that the patients with US uninvolved lymph nodes form a distinct subgroup of melanoma patients. Namely, when patients in the US group were compared to those in the non-US group, we found a statistically significant lower lymph node tumour burden in the US group.

We observed a lower proportion of patients with SN metastases with a diameter of more than 2.0 mm in the US group. The median diameter of SN metastases in the US group was 2.0 mm compared to 6.0 mm in the non-US group. In the logistic regression model, the factors which correlated with the presence of SN metastases with a diameter of more than 2.0 mm were as follows: the preoperative US not preformed, the presence of ulceration and the number of positive SNs.

Furthermore, we found a lower total number of metastatic lymph nodes per patient in the US group of patients with CLND performed in comparison to the non-US group. We also found a lower number of patients with metastatic NSN in the US group. This finding can be explained by the lower proportion of patients with SN metastases with a diameter of more than 2.0 mm in the US group. Namely, the size of the SN metastasis was demonstrated in several studies as being a predictor of the NSN metastases and also as a predictor for survival.^29^–^36^ Hence, due to the lower proportion of patients with SN metastases with a diameter of more than 2.0 mm in the US group, the total number of metastatic lymph nodes and NSN per patient is also lower. As shown by our results, the Breslow thickness, the size of the SN metastasis and the number of SNs removed were significant predictors for the presence of NSN metastases, which is in accordance with previous studies.^29^–^39^

In our study, there was no significant difference in the OS between the US and non-US groups of patients. These results are understandable since the proportion of patients with positive SNs in both groups is small and these are the only patients that could theoretically benefit from a preoperative US. However, when we compared only those patients with positive SNs, the patients in the US group had a significantly better OS than those in the non-US group, with a 5-year OS of 68% compared to a 5-year OS of 43%. Similar results were demonstrated in a study by Voit and colleagues who compared the OS of patients with SN metastases and a positive preoperative US of regional lymph nodes to those with SN metastases and a negative preoperative US.^40^ In their study, patients with SN metastases and negative preoperative US had significantly better OS than those with SN metastases and positive preoperative US, with a 5-year OS of 71% compared to a 5-year OS of 53%. In our study, the patients with lymph node metastases and positive preoperative US had 41% 5-year OS.

According to these results, a preoperative US can further improve the risk stratification of melanoma patients with metastases in SNs (stage IIIa).^41^

Our findings might also have an implication on the regional treatment of melanoma patients. As only 15% to 30% of patients with positive SNs have an additional disease in NSN, investigators have suggested that CLND might not be necessary in all patients with a positive SNB.^42^–^44^ Since the probability of NSN metastases was reduced in our study in the US group of patients, a preoperative US combined with FNAB should be considered as stratification criteria in randomised trials comparing different regional therapies in SN positive melanoma patients.

In our study we proved that the preoperative US of regional lymph nodes spares some patients from undergoing a SNB. In addition, patients with regional lymph node metastases and a negative preoperative US have a significantly lower tumour burden in comparison to those with only clinically negative regional lymph nodes. Most importantly, this is reflected in a better OS for this particular subgroup of patients.

## Figures and Tables

**TABLE 1:** Patients’, tumour and lymph node characteristics and univariate analysis for patients with negative US, patients with suspicious US and for non-US group

	**Negative US group (n = 343)**	**Suspicious US group (n = 48)**	**Non-US group (n = 316)**	**p**
**Age** (years)	57 (12–93)	56 (10–89)	56 (7–87)	NS
Median (range)				
**Sex**				
M	152 (44.3%)	15 (31.2%)	157 (49.7%)	0.040
F	191 (55.7%)	33 (68.8%)	159 (50.3%)	
**Breslow thickness (**mm)	2.1 (0.5–18.0)	2.3 (0.5–17.0)	2.0 (0.5–13.0)	NS
Median (range)				
**Clark level**				
II	6 (1.8%)	2 (4.2%)	18 (5.6%)	
III	112 (32.7%)	13 (27.1%)	116 (36.7%)	0.030
IV	175 (51%)	23 (47.9%)	120 (38%)	
V	16 (4.7%)	3 (6.2%)	18 (5.7%)	
Missing	34 (9.9%)	7 (14.6%)	44 (13.9%)	
**Ulceration**				
Present	124 (36.2%)	19 (39.6%)	91 (28.8%)	NS
Absent	204 (59.5%)	26 (54.2%)	209 (66.1%)	
Missing	15 (4.4%)	3 (6.2%)	16 (5.1%)	
**Number of SN removed per patients**				
Median (range)	2.0 (1–10)	2.0 (1–10)	2.0 (1–10)	NS
**SNB positive patients**	84/343 (24.5%)	17/48 (35.4%)	59/316 (18.7%)	0.018
**SN metastases size**				
> 2.0 mm	25/84 (38%)	6/17 (35.3%)	32/59 (54.2%)	0.030
> 0.2 mm and ≤ 2.0 mm	46/84 (46.5%)	9/17 (53%)	23/59 (39%)	
≤ 0.2 mm	13/84 (15.5%)	2/17 (11.7%)	4/59 (6.8%)	
Median (range)	1.9 (0.03–25.0)	4.0 (0.07–25.0)	6.0 (0.1–45.0)	< 0.001
**Total number of positive lymph nodes**	1.2 (1–3)	1 (1–5)	1.7 (1–8)	0.020
Median (range)				
**NSN metastases**	11/84 (13%)	4/17 (23.5%)	16/59 (23.7%)	NS

US = ultrasound; SN = sentinel lymph node; SNB = sentinel lymph node biopsy; NSN = nonsentinel lymph nodes

**TABLE 2:** Patients’, tumour and lymph node characteristics and univariate analysis for US and non-US group

	**US group (n = 391)**	**Non-US group (n = 316)**	**p**
**Age** (years)			
Median (range)	57 (10–93)	56 (7–87)	NS
**Sex**			
M	167 (42.7%)	157 (49.7%)	NS
F	224 (57.3%)	159 (50.3%)	
**Breslow thickness** (mm)			
Median (range)	2.1 (0.5–18.0)	2.0 (0.5–13.0)	NS
**Clark level**			
II	8 (2%)	18 (5.6%)	0.008
III	125 (32%)	116 (36.7%)	
IV	198 (50.6%)	120 (38%)	
V	19 (4.8%)	18 (5.7%)	
Missing	41 (10.5%)	44 (13.9%)	
**Ulceration**			
Present	143 (36.6%)	91 (28.8%)	
Absent	230 (58.9%)	209 (66.1%)	0.030
Missing	18 (4.5%)	16 (5.1%)	
**Number of SN removed per patients**			
Median (range)	2.0 (1–10)	2.0 (1–10)	NS
**SNB positive patients**	101/391 (25.8%)	59/316 (18.7%)	NS
**SN metastases size**			0.007
> 2.0 mm	31/101 (30.7%)	32/59 (54.2%)	
> 0.2 mm and ≤ 2.0 mm	55/101 (54.4%)	23/59 (39%)	
≤ 0.2 mm	15/101 (14.9%)	4/59 (6.8%)	
Median (range)	2.0 (0.03–25.0)	6.0 (0.1–45.0)	<0.001
**Total number of positive lymph nodes**			
Median (range)	1.2 (1–5)	1.7 (1–8)	0.008
**NSN metastases**	15/101 (14.8%)	16/59 (23.7%)	0.047

US = ultrasound; SN = sentinel lymph node; SNB = sentinel lymph node biopsy; NSN = nonsentinel lymph nodes

**TABLE 3:** Factors correlated with the presence of SN metastases with a diameter of more than 2.0 mm on univariate and multivariate analysis

**FACTOR**	**p (univariate)**	**p (multivariate)**	**Hazard ratio (HR)**	**95.0% CI for HR**
**Age** (years)				
< 45	0.851	/	/	/
≥ 45				
Sex				
M	0.606	/	/	/
F				
**Site**				
Head and neck	0.456	/	/	/
Trunk				
Extremity				
**Breslow thickness** (mm)				
≤ 1	0.131	/	/	/
1.01–2.00				
2.01–4.00				
> 4.00				
**Clark level**				
II	0.198	/	/	/
III				
IV				
V				
**Ulceration**				
Present	0.023	0.025	2.370	1.116 – 5.031
Absent				
**Number of positive SNs**				
1	0.035	0.017	5.398	1.352 – 21.554
2–3				
> 4				
**Preoperative US**				
Negative	0.001	0.002	1.145	1.051 – 1.247
Not performed				

US = ultrasound; SNs = sentinel lymph nodes; HR = hazard ratio; CI = confidence interval

**TABLE 4:** Factors correlated with the presence of NSN metastases on univariate and multivariate analysis

**FACTOR**	**p (univariate)**	**p (multivariate)**	**Hazard ratio (HR)**	**95.0% CI for HR**
**Age** (years)				
< 45	0.339	/	/	/
≥ 45				
**Sex**				
M	0.822	/	/	/
F				
**Breslow thickness** (mm)				
≤ 1				
1.01–2.00				
2.01–4.00	0.002	0.004	3.014	1.410 – 6.439
> 4.00				
**Clark level**				
II				
III				
IV	0.057	/	/	/
V				
**Ulceration**				
Present	0.898	/	/	/
Absent				
**Preoperative US**				
Negative	0.074	/	/	/
Not performed				
**Number of removed SNs**				
1	0.048	0.040	0.349	0.128 – 0.953
> 1				
**Number of positive SNs**				
1	0.503	/	/	/
2–3				
> 4				
**SN metastasis size (mm)**				
≤ 2.0	0.001	0.003	2.347	1.346 – 4.092
> 2.0				

US indicates ultrasound; SNs, sentinel lymph nodes; HR, hazard ratio; CI, confidence interval
